# A pilot study of 3D tissue-engineered bone marrow culture as a tool to predict patient response to therapy in multiple myeloma

**DOI:** 10.1038/s41598-021-98760-9

**Published:** 2021-09-29

**Authors:** Kinan Alhallak, Amanda Jeske, Pilar de la Puente, Jennifer Sun, Mark Fiala, Feda Azab, Barbara Muz, Ilyas Sahin, Ravi Vij, John F. DiPersio, Abdel Kareem Azab

**Affiliations:** 1 Department of Radiation Oncology, Washington University School of Medicine, 4511 Forest Park Ave, St. Louis, MO 63108 USA; 2 Department of Biomedical Engineering, Washington University, St. Louis, MO USA; 3 Cellatrix LLC, St. Louis, MO USA; 4 Cancer Biology and Immunotherapies Group, Sanford Research, Sioux Falls, SD USA; 5 Department of Medicine, Washington University School of Medicine, St. Louis, MO USA; 6 Division of Hematology/Oncology, The Warren Alpert Medical School, Brown University, Providence, RI USA

**Keywords:** Tissue engineering, Cancer models, Haematological cancer, Tumour heterogeneity

## Abstract

Cancer patients undergo detrimental toxicities and ineffective treatments especially in the relapsed setting, due to failed treatment attempts. The development of a tool that predicts the clinical response of individual patients to therapy is greatly desired. We have developed a novel patient-derived 3D tissue engineered bone marrow (3DTEBM) technology that closely recapitulate the pathophysiological conditions in the bone marrow and allows ex vivo proliferation of tumor cells of hematologic malignancies. In this study, we used the 3DTEBM to predict the clinical response of individual multiple myeloma (MM) patients to different therapeutic regimens. We found that while no correlation was observed between in vitro efficacy in classic 2D culture systems of drugs used for MM with their clinical efficacious concentration, the efficacious concentration in the 3DTEBM were directly correlated. Furthermore, the 3DTEBM model retrospectively predicted the clinical response to different treatment regimens in 89% of the MM patient cohort. These results demonstrated that the 3DTEBM is a feasible platform which can predict MM clinical responses with high accuracy and within a clinically actionable time frame. Utilization of this technology to predict drug efficacy and the likelihood of treatment failure could significantly improve patient care and treatment in many ways, particularly in the relapsed and refractory setting. Future studies are needed to validate the 3DTEBM model as a tool for predicting clinical efficacy.

## Introduction

Personalized medicine is the use of the patient’s own tumor genotype and/or phenotype to create the most appropriate therapeutic choice for individual patients. While precision cancer medicine studies have mainly focused on genomics, most patients with cancer who receive genomic testing have limited benefit from this strategy^1^. This is due to the fact that drug sensitivity and resistance depend not only on genomics, but also on epigenetic factors, and most importantly the interactions with tumor microenvironment (TME)^2^. 2D classic tissue culture models lack significant biological features of the TME such as oxygen gradients (tumor hypoxia) and drug gradients, which have critical effect on the sensitivity of cancer cells to therapy in different hematologic malignancies such multiple myeloma (MM)^3^, leukemia^4^, and lymphoma^5,6^. While synthetic 3D models such as polymeric matrices exhibit advantages compared to classic 2D cultures, they utilize non-physiological materials that may change the biological properties of the tumor cells^7^. In addition, the presence of accessory cells found in the TME, aside from the tumor cells, was shown to play a critical role in the TME-induced resistance^2,8^. Therefore, a patient-derived 3D model of primary tumor cells, including other accessory cells, without exogenous polymers, that truly recapitulates the TME is warranted.

**Figure 1:**
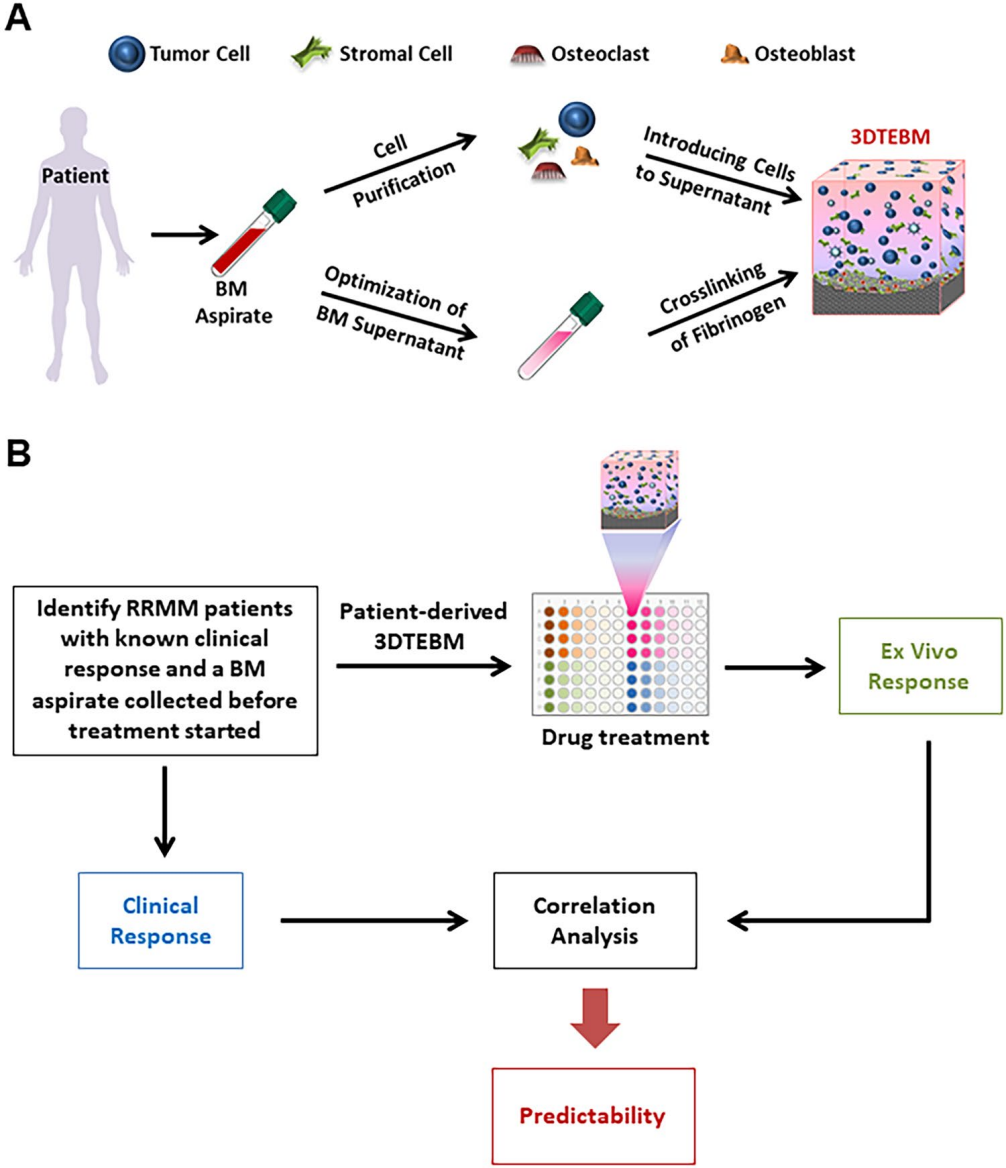
Novel patient-derived 3D Tissue-Engineered Bone Marrow (3DTEBM) used to predict clinical efficacy in individual cancer patients for personalized medicine. (**A**) The 3DTEBM includes all the accessory and primary cancer cells found in the bone marrow (BM), as well as growth factors, enzymes, and cytokines naturally found in the TME which better recapitulates the BM niche found in patients. (**B**) Clinical workflow for a retrospective study testing 3DTEBM predictability for multiple myeloma (MM) patient clinical response.

We have previously created a patient-derived 3D tissue-engineered bone marrow (3DTEBM) culture model, which is predominantly derived from BM aspirates of patients with hematologic malignancies (Fig. 1A). It utilizes autologous BM supernatant to create the 3D matrix, in which we incorporate primary cancer cells, along with all the other accessory cells in the BM microenvironment, without the addition of exogenous materials^9^. The BM cellular and non-cellular constituents recapitulate the malignant TME enabling primary cells progression *ex vivo*^9^. In addition, the hydrogel-like 3D structure derived from the crosslinking of endogenous fibrinogen results in a “soft” culture compared to other synthetic polymers, which better recapitulates the BM tissue^2,10^. Moreover, the 3D structure recreated oxygen gradients, resembling tumor hypoxic BM niche. It was demonstrated that the 3DTEBM was able to promote biological effects which were not demonstrated in regular 2D culture^2,10–12^, and allow the survival and proliferation of freshly isolated or frozen primary cancer cells ex vivo*,* in both MM^10,12^ and leukemia^11^.

The 3DTEBM model was shown to mimic the malignant BM condition in several hematologic malignancies including MM^9^, acute myeloid leukemia, chronic myeloid leukemia, and chronic lymphocytic leukemia^11^, allowing primary cell progression ex vivo and better representation of drug resistance/sensitivity^7,9,11^. Therefore, the 3DTEBM arises as a unique model for ex vivo drug screening and predictive tool to determine clinical efficacy in individual cancer patients for personalized medicine.

MM is the cancer of plasma cells within the BM and represents the second most common hematologic malignancy^13^. Although therapeutic options have significantly broadened over the years, the disease is challenged by frequent relapses. Relapsed/refractory MM (RRMM) often becomes non-responsive to previous lines of treatment and has significantly poorer survival outcome^14^. Physicians are faced with a difficult task to choose a right treatment regimen for RRMM patients^15^. In this study, we aimed to conduct a retrospective study that tests the ability of the ex vivo 3DTEBM platform to predict the clinical response in individual MM patients, to help decision-making process for RRMM. 3DTEBM cultures were developed for each patient from BM biopsies obtained before the start of clinical treatment. Cultures were treated ex vivo with the same treatment regimen each patient received clinically. Finally, ex vivo responses in the 3DTEBM were correlated to clinical responses, to test overall predictability (Fig. 1B). We hypothesized that the 3DTEBM will be able to predict clinical responses of RRMM patients.

## Results

**Figure 2:**
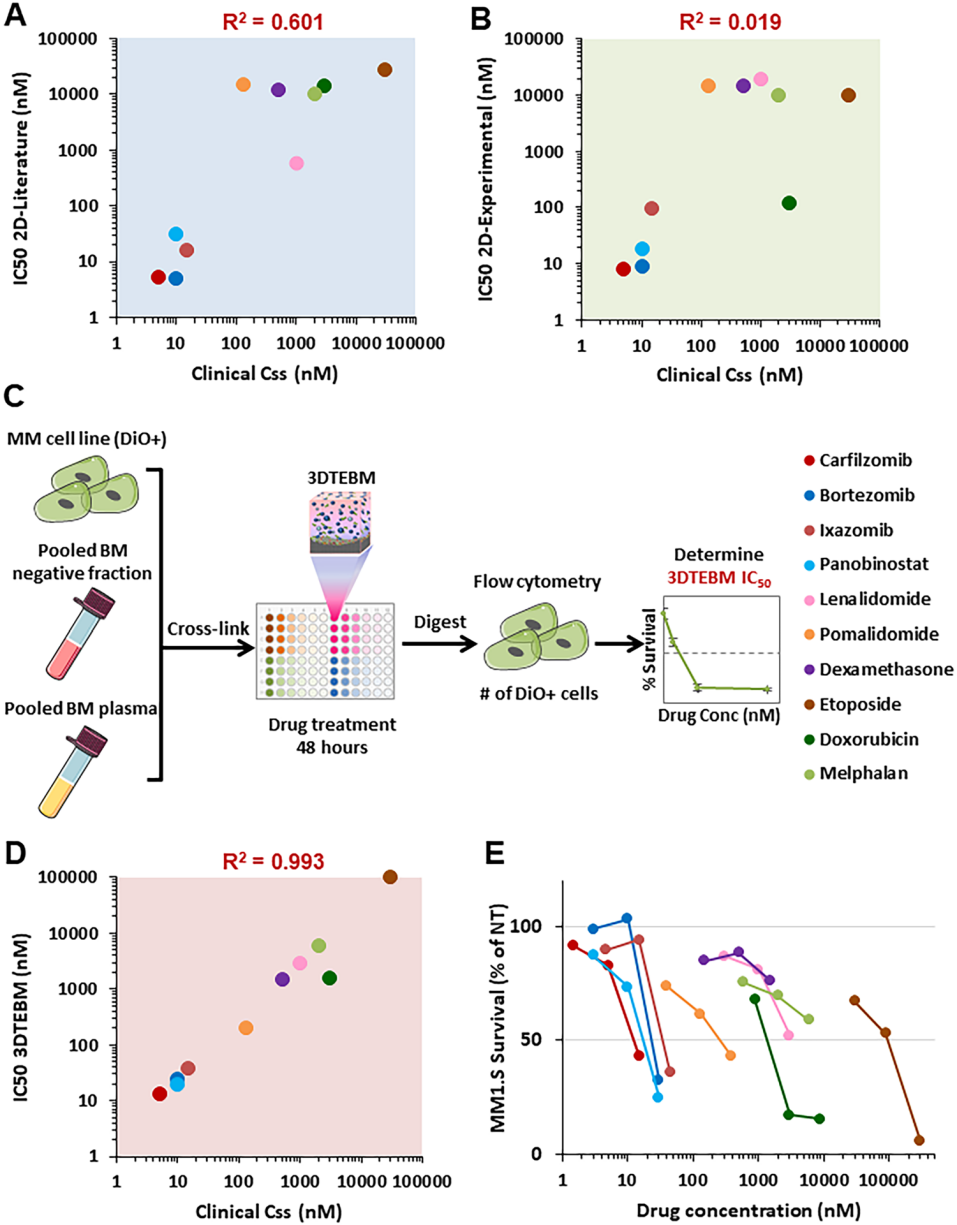
The 3DTEBM efficaciously correlates between half maximal inhibitory concentration (IC50) and clinical steady state plasma concentration (Css) values of MM drugs. IC50 values determined by (**A**) 2D literature research and (**B**) 2D experimental studies both correlated poorly with clinical Css values. (**C**) Schematic of experimental procedure for determining 3DTEBM IC50 in cell lines. (**D**) IC50 values determined by 3DTEBM correlated well with clinical Css values. (**E**) 3DTEBM dose response curves for all 10 drugs tested in MM1.S cells. Correlation coefficients (R^2^) were calculated from linear regression fitting.

Despite the ability of therapeutic agents to eradicate MM in vitro in classic 2D culture models, there is often little correlation with clinical activity in patients. To demonstrate this discrepancy between drug efficacy in laboratory settings and clinical outcomes, we compared the in vitro efficacious concentration to the clinical efficacious concentrations of 10 drugs used for the treatment of MM (Table 1). We defined the efficacious clinical concentration as the steady state plasma drug concentration (Css) reached after administration of clinically effective doses. We performed a literature search and determined Css values based on pharmacokinetic data from Phase 1 and/or Phase 2 clinical trials (Table 1). Next, we defined the efficacious in vitro concentration as the half-inhibitory concentration (IC_50_) at 48 h for each drug in MM cell lines. The IC_50_ values for the 2D classic tissue culture system was determined by both literature search and experimental study. With these information, we calculated the correlation between the 2D IC50 values and clinical Css values. We found that there was no correlation between the in vitro 2D IC_50_ and the clinical Css values, represented by a correlation coefficient of R^2^ = 0.601 for literature studies (Fig. 2A) and R^2^ = 0.019 for experimental study (Fig. 2B).

**Table 1:** Names, concentrations, and literature for each of the drugs used.

MM drug	Literature Css (nM)		2D Literature IC_50_ (nM)	
Carfilzomib	5	^16–18^	5.3	^19–21^
Bortezomib	10	^22^	5	^19,23–25^
Ixazomib	15	^26,27^	15.8	^24,28^
Panobinostat	10	^29,30^	31.2	^31,32^
Lenalidomide	1000	^33,34^	600	^35–37^
Pomalidomide	130	^38^	14,773.7	^39,40^
Dexamethasone	500	^41^	11,900.7	^42–45^
Etoposide	30,000	^46,47^	28,000.1	^48–50^
Doxorubicin	3000	^51^	14,536.8	^52–54^
Melphalan	2000	^55,56^	10,000	^57–59^

We then determine the IC_50_ values of the same drugs in the 3DTEBM culture system, and their correlation with clinical Css values. MM cells lines were cultured within the 3DTEBM in combination with MM patient BM microenvironment, and the MM cell survival was determined at 48 h (Fig. 2C). In contrast to 2D tissue cultures, we found that the IC_50_ values in the 3DTEBM directly correlated with the clinical Css, represented by a correlation coefficient of R^2^ = 0.993 (Fig. 2D). The individual drug’s dose response curves are also shown (Fig. 2E and Supp Fig. 1).

Next, we conducted a retrospective clinical trial to determine if the 3DTEBM platform is able to predict each patient’s clinical response by recreating the same treatment regimen ex vivo. 19 RRMM patients with known clinical responsiveness to the regimen they received were identified. The patients had a mix of responsiveness: patients with partial response (PR), very good partial response (VGPR) and/or complete remission (CR) were categorized as “responsive”, while patients with stable disease and/or progressive disease (PD) were categorized as “non-responsive”. Patient demographics for the primary MM samples used are shown in Table 2.

**Table 2:** Clinical characteristics of patients with multiple myeloma.

Clinical characteristics	All patients (n = 19)
Median Age	61.8 years (range 46–82)
Gender	Value (%)
Male	7 (37)
Female	12 (63)
Race	Value (%)
African American	2 (11)
White	17 (89)
Treatment Status	Value (%)
Relapse/Progression	19 (100)

**Figure 3:**
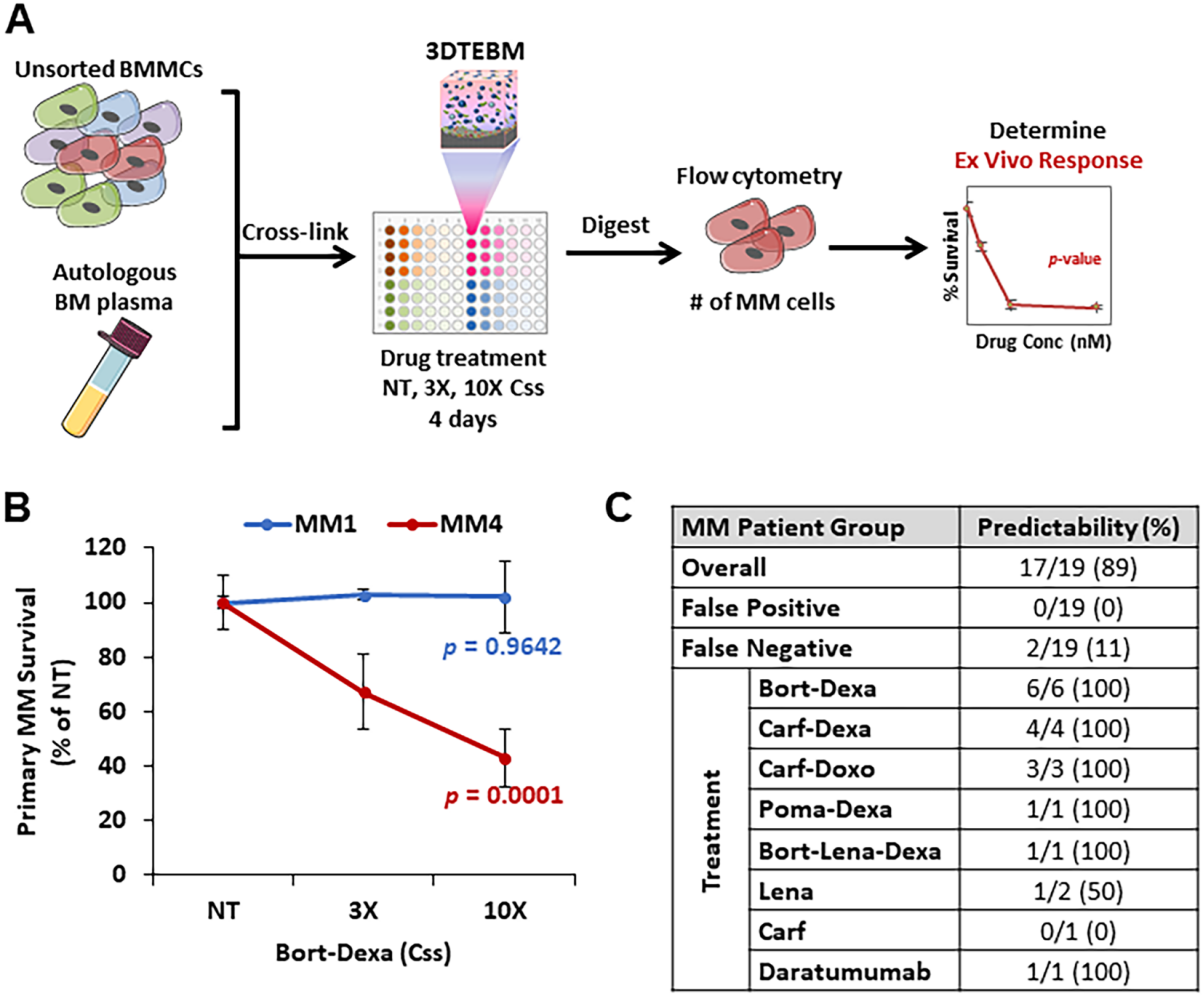
Retrospective predictability for MM patient cohort. (**A**) Schematic of experimental procedure for determining ex vivo response in 3DTEBM utilizing patient unsorted bone marrow mononuclear cells (BMMCs) and autologous BM plasma. (**B**) Example primary MM cell survival for a responsive and a non-responsive MM patient sample. Statistical significance is analyzed by single-factor ANOVA; responsive if p < 0.05. (**C**) Overall predictability of patient clinical response for each drug therapy using the 3DTEBM platform.

We developed 3DTEBM cultures using individual patient’s BM samples collected prior to the start of their prescribed treatment regimen. Samples were treated for 4 days with the respective regimen at increasing concentrations, and primary cell survival was determined as % of untreated (Fig. 3A). The ex vivo response was given back to the clinical teams as “responsive” or ”non-responsive”. To demonstrate the range of ex vivo response we observe in the 3DTEBM, primary cell survival curves resulting from 3DTEBM cultures derived from two MM patients are presented (Fig. 3B), each of which were treated with the same drug regimen. MM Patient 4 did not respond (p-value of 0.9642) to the combination treatment of bortezomib and dexamethasone, while MM Patient 4 responded to the treatment (p-value of 0.0001). Additionally, survival curves for all 19 patients representing their ex vivo response are shown in Supplemental Fig. 2. The clinical teams correlated the ex vivo response for each patient with the clinical response.

Finally, we developed a summary, highlighting the predictability of patient response by the 3DTEBM ex vivo, based on the drug regimen that the patient received clinically. The 3DTEBM model was able to predict the response in 89% of the MM patient cohort (Fig. 3C). Particularly, it was able to predict 100% of the non-responsive cases in MM, and 75% in MM responsive cases. A more detailed description of the clinical responses, and responses predicted by 3DTEBM for each patient sample are listed in Table 3.

**Table 3:** Clinical responses and predictability of 3DTEBM.

Patient	Treatment	3DTEBM		Clinical Response	Predictive
		P-value	Response		
1	Bort-Dexa	0.9642	Not sensitive	PD	Yes
2	Bort-Dexa	< 0.0001	Sensitive	PR	Yes
3	Bort-Dexa	0.4629	Not sensitive	PD	Yes
4	Bort-Dexa	0.0001	Sensitive	PR	Yes
5	Bort-Dexa	< 0.0001	Sensitive	PR	Yes
6	Bort-Dexa	0.3522	Not sensitive	PD	Yes
7	Carf-Dexa	0.0008	Sensitive	VGPR	Yes
8	Carf-Dexa	0.7661	Not sensitive	PD	Yes
9	Carf-Dexa	0.1092	Not sensitive	PD	Yes
10	Carf-Dexa	0.4130	Not sensitive	PD	Yes
11	Carf-Doxo	0.0038	Sensitive	PR	Yes
12	Carf-Doxo	0.1006	Not sensitive	PD	Yes
13	Carf-Doxo	0.5779	Not sensitive	PD	Yes
14	Bort-Lena-Dexa	0.5334	Not sensitive	PD	Yes
15	Lena	0.3361	Not sensitive	PR	No
16	Lena	0.0236	Sensitive	VGPR	Yes
17	Carf	0.1405	Not sensitive	CR	No
18	Poma-Dexa	0.6661	Not sensitive	PD	Yes
19	Daratumumab	0.5263	Not sensitive	PD	Yes

## Discussion

The majority of clinical trial failures are due to a lack of drug efficacy (52%)^60^. The traditional 2D culture system does not recapitulate the TME nor the clinical outcomes. In this study, we demonstrated that there was no correlation between the in vitro IC_50_ values in classic 2D culture systems and the clinically efficacious concentrations. These findings could partially explain the discrepancies between drug efficacy in laboratory settings and the unsatisfactory clinical outcomes in hematologic malignancies. In contrast, IC_50_ values obtained in the 3DTEBM showed direct correlation with clinically efficacious concentrations. This suggests that, unlike regular culture systems, the 3DTEBM platform provides a pathophysiologically relevant model that could be used for drug development and help predict drug efficacy in individual patients.

Recent studies in the field have suggested a variety of ex vivo approaches to identify drugs with clinical efficacy in hematologic malignancies^61–64^. Despite intensive efforts in the field, reliable, cost-effective and simple ex vivo model that is applicable in the clinical practice for drug efficacy prediction is still lacking^62^. A reliable method predicting drug efficacy and the likelihood of treatment failure could significantly improve patient care and treatment in many ways particularly in relapsed/refractory setting. Potential benefits will include but not limited to avoiding the toxicity and reducing the costs of therapies that prove to be ineffective.

In this retrospective study, we demonstrated that the 3DTEBM technology was able to predict clinical therapeutic response with 89% accuracy. These results suggest the 3DTEBM as a feasible platform for predicting therapeutic responses in MM with a high predictive accuracy within a clinically actionable time frame. Routine bone marrow biopsy, in addition to its diagnostic value, can be now utilized in the 3DTEBM as a treatment decision-making tool. Such platforms can provide precise clinical insight about the efficacy of different treatment plans in a timely manner (less than a week), and assist physicians to propose the best choice of therapy for their individual patients.

3DTEBM is emerging as a promising personalized medicine platform for prediction of clinical responses to therapy in individual RRMM patients, not only because of its ability to allow proliferation of patient-derived primary tumor cells and its ability to mimic patient’s own pathophysiologic BM TME ex vivo, but also because of its accuracy, reproducibility, short turnaround time, high-throughput potential, and low-cost. Future prospective studies are needed to validate these significant findings by expanding clinical sample size, and testing the ability of the 3DTEBM to prospectively predict effective treatment for improved response in hematologic malignancies.

## Methods

### Materials and reagents

Lipophilic cell tracer (DiO) was purchased from Invitrogen (Eugene, OR). All antibodies used for flow cytometry were purchased from BD Biosciences (San Jose, CA) unless otherwise noted. Red Blood Cell (RBC) lysis buffer was purchased from Biolegend (San Diego, CA). All chemicals were purchased from Millipore Sigma (Burlington, MA). All drugs were purchased from Selleck Chemicals (Houston, TX), except Daratumumab (Janssen, Beerse, Belgium), which was generously provided to us by the pharmacy at Washington University. MM cell lines were obtained from American Type Culture Collection (Manassas, Virginia).

### Patient primary BM samples

MM pre-treated patient samples were collected under informed consent, in concordance with the Institutional Review Boards (IRBs) of Washington University in St. Louis School of Medicine (IRB protocol number 201102270). All studies were in accordance with the Declaration of Helsinki. Plasma was isolated by centrifugation, and BM mononuclear cells (BMMCs) were isolated by RBC lysis, as described before^65^. To acquire BM negative fraction cells, BMMCs from MM aspirates were depleted of CD138 by magnetic beads, as previously described^66^.

### Cell culture and 3DTEBM culture

MM cell lines, MM.1S, H929, and OPM2, were purchased from American Type Culture Collection (Rockville, MD). All cell lines were cultured in RPMI-1640 media (Corning, Corning, NY) supplemented with 10% fetal bovine serum, 2 mmol/L L-glutamine, 100 μg/mL penicillin, and 100 μg/mL streptomycin. All cells were cultured at 37 °C and in 5% CO2 in a NuAire water jacket incubator (Plymouth, MN).

3DTEBM were prepared as previously described^9^. Briefly, cells of interest were plated in 96-well plate in a mixture of BM supernatant, RPMI media, tranexamic acid, and calcium chloride (10 mg/mL). After the 3D matrix cross-links, they were supplemented with media on top and cultured at 37 °C and 5% CO_2_. At the end of the experiment, matrix were digested by type I collagenase, CountBright absolute counting beads (ThermoFisher) were added to each well, and samples were filtered using a 35 um nylon mesh filter and subsequently analyzed using flow cytometry (MACSQuant Analyzer 16, Miltenyi Biotec, Bergisch Gladbach, Germany).

### Correlation of 2D and 3DTEBM IC50 values with clinical Css values

The drugs used for the correlation study were carfilzomib, bortezomib, ixazomib, panobinostat, lenalidomide, pomalidomide, dexamethasone, etoposide, doxorubicin, daratumumab, and melphalan for MM. Summary of 2D-Literature, 2D-Experimental, and 3DTEBM IC_50_ values, as well as clinical Css values for each drug are shown in Table 1.

The steady state plasma drug concentration (Css) was determined based on pharmacokinetic data from Phase 1 and/or Phase 2 clinical trials. For 2D-Literature IC_50_ values, 48 h IC_50_ values of MM cell lines from at least two studies were averaged. For 2D-Experimental IC50 values, MM cells (MM.1S, H929, and OPM2) were seeded at 50,000 cells per well in 96-well plate and treated with or without incremental concentration for respective drugs for 48 h. Cell survival was determined by MTT assay as previously described^67^. Briefly, MTT solution was added to the cells for 3 h, then the stop solution was added to dissolve the formazan crystals overnight. Wells were read with SpectraMax i3 multimode microplate spectrophotometer (Molecular Devices, San Jose, CA) at 570 nm.

3DTEBM IC_50_ values were found by increasing the concentration of each drug inside and on top of the 3DTEBM cultures for three different cell lines (MM.1S, H929, and OPM2). 3DTEBM were prepared with DiO-labeled MM cells (50,000/well), BM negative fraction (50,000/well, pooled from 5 RRMM patients), and BM plasma (pooled from 5 RRMM patients), and RPMI media mixed with treatment. After crosslinking of 3DTEBM for 2 h, the matrix was supplemented with 10% plasma in RPMI media on top. Drug treatment was performed at 0.3X, 1X, 3X, and 10X the Css concentration in the 3DTEBM for 48 h. At the end of the experiment, 3D matrix were digested by collagenase and counting beads were added to each well. Each sample was filtered and analyzed using flow cytometry for the number or the DiO-labeled cancer cells, and normalized to the number of beads. Dose escalation curves were graphed, and the IC_50_ concentrations for each drug in the three cell lines were determined and averaged to find the final 3DTEBM IC_50_ values.

### Retrospective MM patient 3DTEBM responses

Patient BM samples (unsorted BMMCs and BM plasma) were collected before the start of the clinical treatment regimen. Samples were provided to Cellatrix, along with the treatment regimen that each patient received after the BM biopsy; however, Cellatrix was blinded to the clinical outcomes. Cellatrix treated each patient sample with the corresponding regimen in the 3DTEBM and provided the ex vivo response back to the clinical team (as demonstrated in Fig. 1B).

Patient 3DTEBM were developed by seeding 1 × 10^5^ BMNCs per well with patient’s autologous BM plasma and treated with or without the corresponding drug regimen in increasing concentrations (3X and 10X of Css of individual drugs). Following four days of treatment, cultures were digested to retrieve the cells for flow cytometry analysis. Samples were stained with CD3, CD14, CD16, CD19 and CD123 (all of which were FITC) in addition to CD38 (APC), according to our previously published MM detection method^68^. For flow analysis, live cells were first gated as FSC-hi. Then, MM cell survival was determined by number of FITC-/APC+ cells, normalized to counting beads, and the effect of the drug treatment was calculated as percent of untreated control.

Ex vivo response was analyzed by ANOVA and the responsiveness was provided to the clinical team. The clinical response was determined by the clinical team after a cycle of respective regimen, defined according to the Criteria of the International Myeloma Working Group^69^. The clinical team correlated the ex vivo response with the clinical response for each patient.

### Statistical analyses

All patient sample data experiments were expressed as means ± standard deviation. Sample size was estimated using published guidelines. 3DTEBM experiments were performed in quadruplicates. Residuals were used to analyze data normality, and we examined the expected variance by analyzing the variance similarity across groups. Statistical significance for comparing ex vivo response of primary cells across different concentrations was analyzed using a one-way analysis of variance (ANOVA). P-values < 0.05 were considered statistically significant. Correlation between IC_50_ and Css values determined by linear regression fitting.

## Supplementary Information

Supplementary Information.

## Data Availability

All data generated are available upon request.
