# Supplementary material for: A pilot study of 3D tissue-engineered bone marrow culture as a tool to predict patient response to therapy in multiple myeloma
Source: Sci Rep. 2021 Sep 29;11:19343. doi: 10.1038/s41598-021-98760-9 (PMC8481555; doi:10.1038/s41598-021-98760-9)

3D Tissue-Engineered Bone Marrow Culture Predicts
Patient Response to Drugs in Multiple Myeloma

Supplemental Figures

SUPPLEMENTAL FIGURE LEGENDS

Supp Figure 1. 3DTEBM dose response curves for individual drugs on MM1.S cells.

Supp Figure 2. Primary MM cell survival for the tested treatment regimen in individual patients. Statistical significance is analyzed by single-factor ANOVA; red indicates sensitive ($p<0.05$) and blue indicates not sensitive ($p>=0.05$) to treatment.

Supp Figure 1

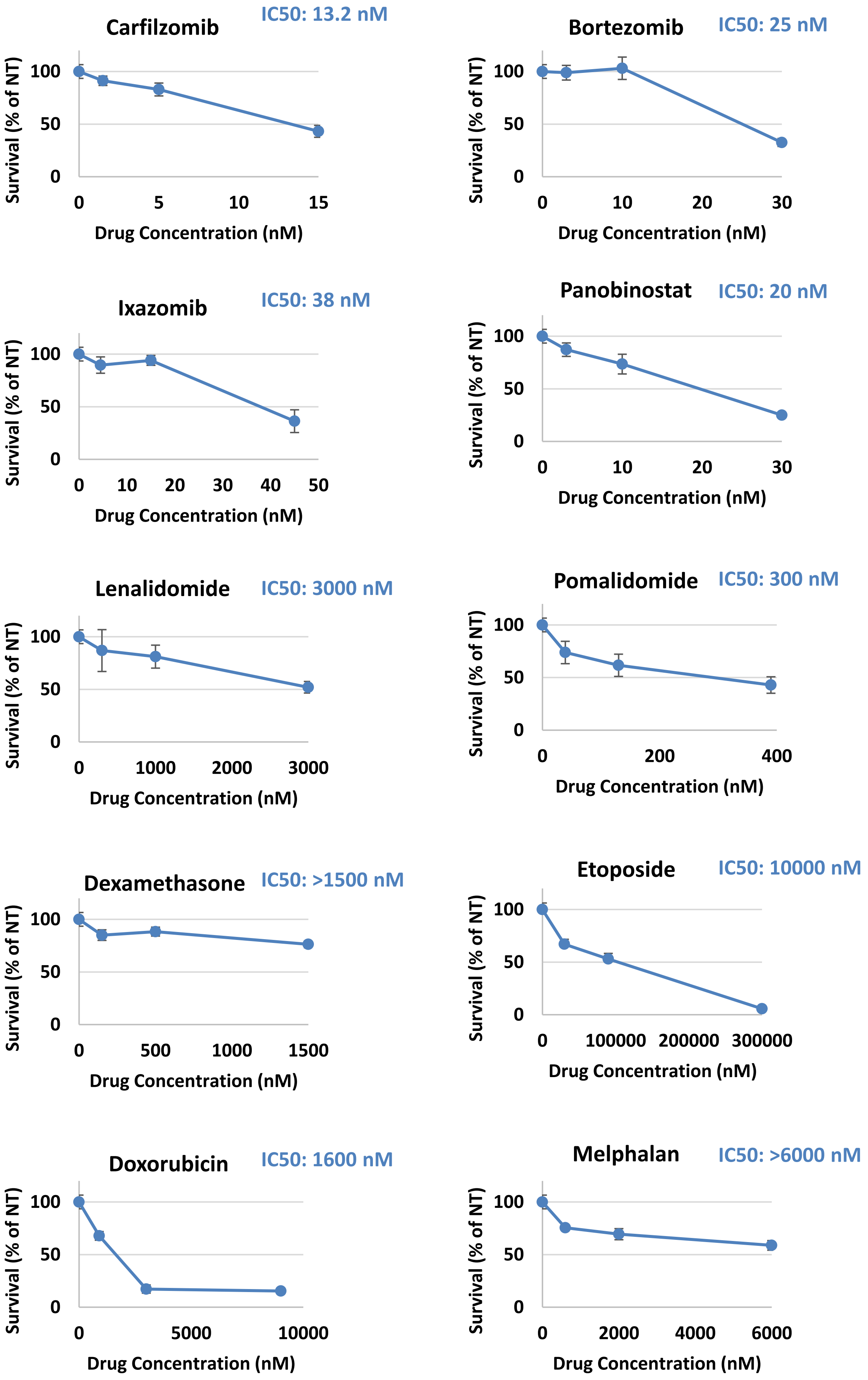

Supp Figure 2

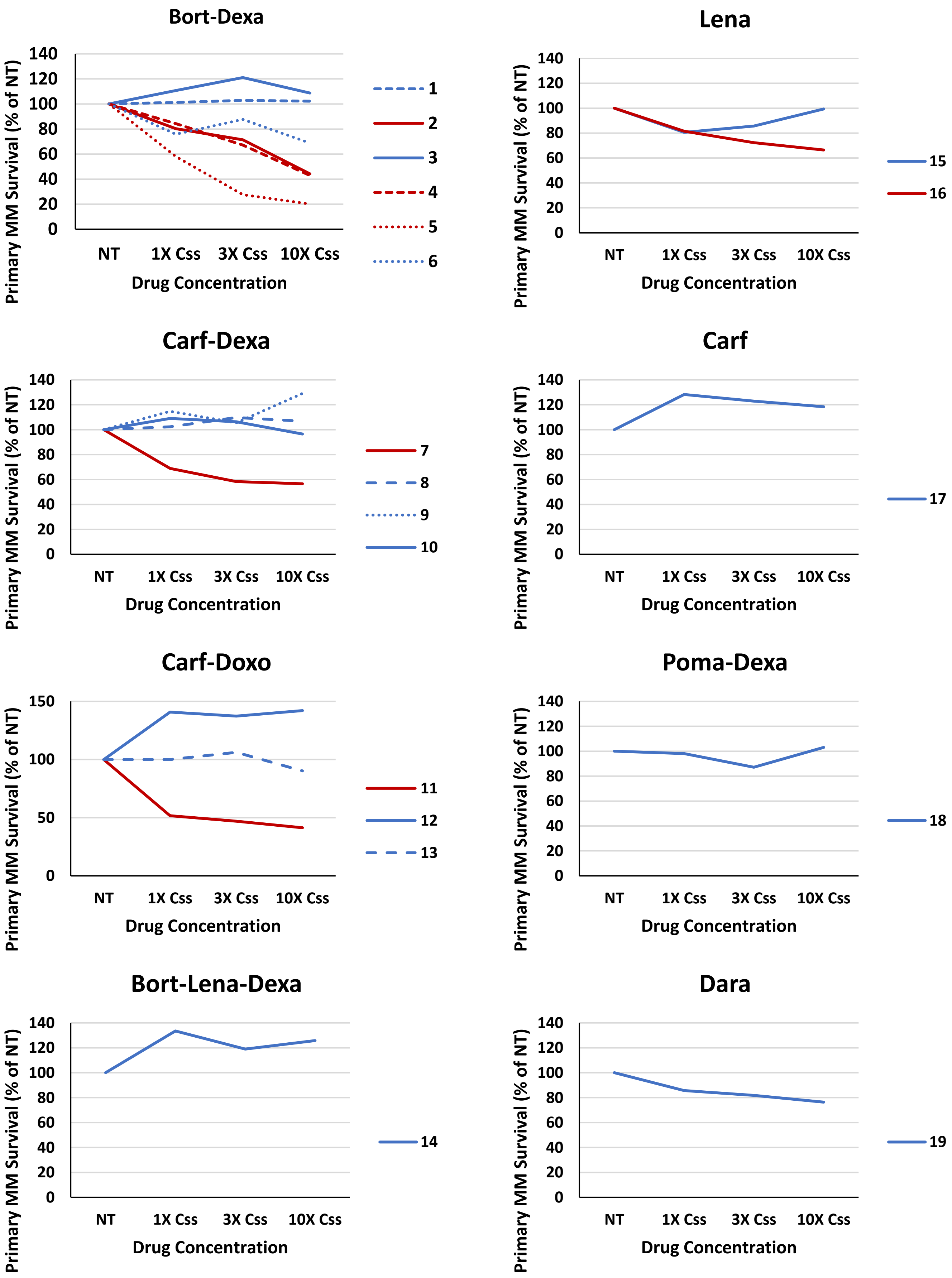

Supplement: Supplementary file 1 — Supplementary Information. [file 41598_2021_98760_MOESM1_ESM.pdf]
